# Electrochemical Corrosion Behavior of MIG-Welded 7N01-T4 Aluminum Alloy by ER5356 and ER5087 Welding Wires

**DOI:** 10.3390/ma15103737

**Published:** 2022-05-23

**Authors:** Ping Wei, Mingfang Wu, Dashuang Liu, Ziqiang Zhao, Yun Liang, Zhihui Dong

**Affiliations:** 1 School of Material Science and Engineering, Jiangsu University of Science and Technology, Zhenjiang 212003, China; semwp@126.com (P.W.); echo2015@163.com (M.W.); wuxizhaoziqiang@163.com (Z.Z.); ly18852894156@163.com (Y.L.); 13526825089@163.com (Z.D.); 2 School of Material Science and Engineering, Hefei University of Technology, Hefei 230009, China

**Keywords:** 7N01-T4 aluminum alloy, MIG, ER5356 welding wire, ER5087 welding wire, electrochemical corrosion

## Abstract

7N01-T4 aluminum alloy plates were welded by the metal inert gas (MIG) welding method, with ER5087 and ER5356 welding wires, respectively. The electrochemical corrosion behavior of the weld zones in the two kinds of welded joints using 3.5 wt.% and 5 wt.% NaCl solutions were investigated by polarization curve, electrochemical impedance spectroscopy (EIS), scanning electron microscope (SEM), and laser confocal scanning microscope (LCSM). The results indicated the better corrosion resistance of the weld zone in the ER5356 welded joint than that in the ER5087 welded joint, which was related to the different contents of Mn and Zn elements and the distribution of precipitates for the weld zones in the two kinds of welded joints. Based on the LSCM of the weld zones, the maximum depth (*d_max_*) of corrosion pits for the weld zone in the ER5356 welded joint was lower than that in the ER5087 welded joint when immersed in the same NaCl concentrations. The *d*_max_ of the corrosion pit of the weld zone in the ER5356 welded joint using the 5 wt.% NaCl solution was 78.5 ± 0.96 μm, which was much bigger than that using the 3.5 wt.% NaCl solution. For the weld zone in the ER5087 welded joint with 5 wt.% NaCl solution, more Cl^-^ was adsorbed onto the active surface of weld zones, which accelerated the corrosion, resulting in the corrosion mechanism from pitting to intergranular corrosion.

## 1. Introduction

The Al-Zn-Mg alloy has seen wide acceptance in the manufacturing field of high-strength and lightweight structures, such as high-speed trains, marine ships, aerospace, and other fields. As a typical Al-Zn-Mg alloy, 7N01 aluminum alloy has excellent extrusion property and good welding performance as an ideal lightweight material widely used in the body structure of high-speed trains [1,2,3].

Welding wire is a critical factor affecting the composition, microstructure, hot crack resistance of weld metal and base metal near the weld, corrosion resistance, and mechanical properties of welded joints. The common filler wires for 7N01 aluminum alloy welding mainly include ER5356 (Al-Mg) and ER5087 (Al-Mg) welding wires. Al-Mg welding wire increases the content of magnesium in the weld and forms strengthening phase Al_8_Mg_5_ [4,5]. In comparison with ER5356 welding wire, ER5087 welding wire contains trace Zr and more Mn elements. Zr and Al peritectic reaction occurs to form dispersed and coherent with the matrix Al_3_Zr, which provides the basis for heterogeneous nucleation to promote the formation of fine equiaxed crystals, so as to improve the strength of weld metal [6,7]. Some researchers have studied the influence of ER5356 and ER5087 welding wires on the microstructure and mechanical properties of 7N01 aluminum alloy welded joints. Xie et al. [8] found that the tensile strength and elongation of the ER5087 welded joint were greater than those of the ER5356 welded joint. This is attributed to refining the structure by adding Zr element to the formation of Al_3_Zr. Liu et al. [9] found the porosity and the eutectic melting in the heat affected zone led to crack initiation for ER5087 and ER5356 welded joints during the fatigue process, respectively. However, there are few reports comparing the effect of ER5356 and ER5087 welding wires on the corrosion properties of 7N01 aluminum alloy joint. A 7N01 aluminum alloy welded joint experienced the rapid hot and cold process, the local microstructure changed complex, the precipitated phase produced unbalanced dissolution and precipitation behavior, and redistributed in the boundary and grain boundary, resulting in the non-uniformity structure of a welded joint. Thus, the corrosion resistance decreased [10,11]. Moreover, 7N01 aluminum alloy has corrosion sensitivity in humid and chlorine environments [12,13,14]. Long term exposure of the 7N01 aluminum alloy body of a high-speed train to atmospheric environment and polluted anions in urban and coastal areas will cause corrosion damage, leading to deterioration of the structure and performance. So, it is of practical significance to study the corrosion behavior of 7N01 aluminum alloy welded joints to ensure the safe operation of high-speed trains.

In this paper, 7N01-T4 aluminum alloy was MIG welded with ER5087 and ER5356 welding wires, respectively. Electrochemical methods were used to study the corrosion behavior of the weld zones of ER5356 and ER5087 welded joints in 3.5 wt.% and 5 wt.% NaCl solutions, respectively. The morphology and the *d*_max_ of corrosion pit of the weld zones observation were investigated.

## 2. Experimental Procedure

### 2.1. Experimental Materials

7N01-T4 aluminum alloys were used as the base materials in the form of 12 mm thick plates. The filling wires were ER5087 and ER5356 alloy with a diameter of 1.6 mm. The chemical compositions of 7N01-T4 aluminum alloy, ER5356 and ER5087 welding wires used in the experiment are listed in Table 1.

### 2.2. Welding Process

The 7N01-T4 aluminum alloy was butt joined using MIG welding. High-purity argon gas (99.999%) was used as the shield gas. A “V-shape” groove was made by an angle of 70°, and the root face was 2 mm. The welding direction was perpendicular to the rolling direction. The welding process was divided into three passes. The welding process parameters are given in Table 2.

### 2.3. Electrochemical Corrosion Test and Microstructural Observation

**Figure 1:**
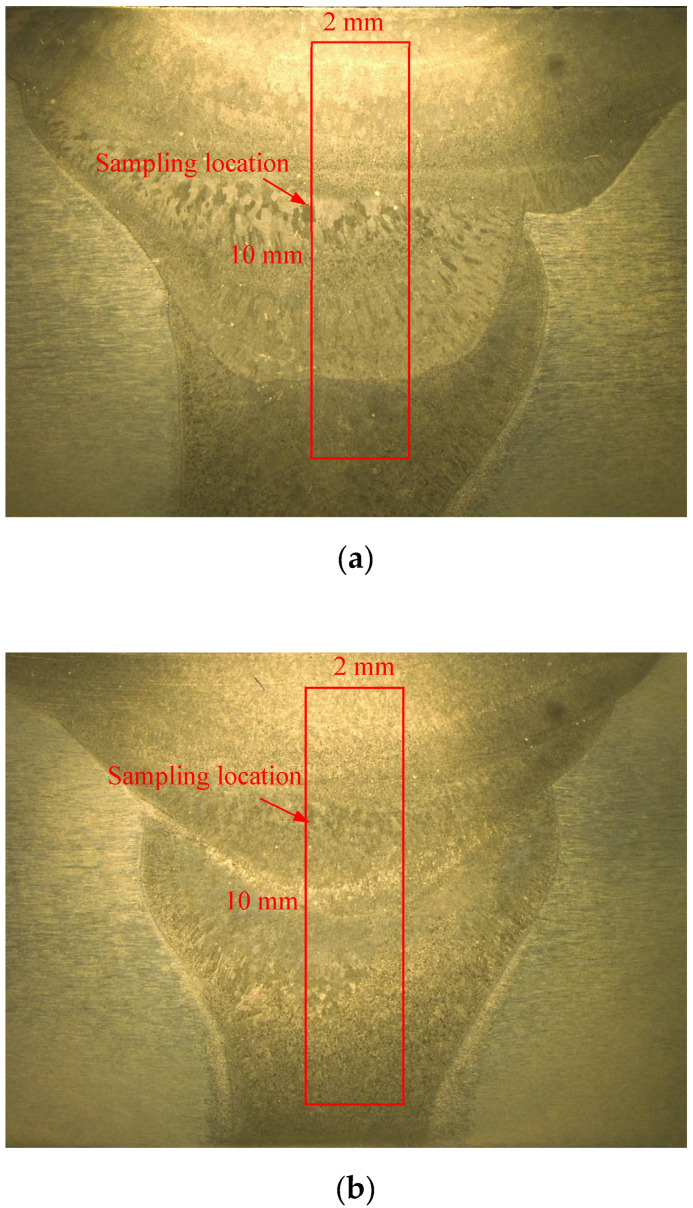
Sampling location in different welded joints for electrochemical corrosion test, (**a**) ER5087 welded joint, (**b**) ER5356 welded joint.

Figure 1 shows the sampling location in ER5087 and ER5356 welded joints for the electrochemical corrosion test. Electrochemical experiments of the two kinds of weld zones were carried out on Coster CH350S electrochemical workstation in 3.5 wt.% and 5 wt.% NaCl solutions, respectively.

Table 3 shows the abbreviation of samples. Three electrode system devices were used in the test. A saturated calomel electrode (SCE) was adopted as the reference electrode. Pt sheet was used as the auxiliary electrode and 1 cm^2^ electrode area was served as the working electrode. The samples were immersed in the NaCl solutions for 1 h before testing the open circuit potential (OCP). The measurement time of the OCP was 20 min to make the electrode system reach a stable state. The electrochemical impedance test frequency ranged from 10^5^ to 10^−2^ Hz, and the amplitude of AC signal imposed was 10 mV. Zview software was used to analysis the EIS measurements. The experiment of potentiodynamic polarization was performed after the EIS test. The scanning range of the potential was ±50 mV (relative to the OCP) with a scan rat 0.5 mV/s. The corrosion potential (*E*_corr_) and corrosion current density (*I*_corr_) were obtained by Tafel-type fit from the polarization curves. All electrochemical measurements were repeated three times to ensure the accuracy of test results.

The micromorphology was observed by SEM (JSM-6480). The energy disperse spectroscopy (EDS) in SEM was used to analyze the chemical composition. The depth of corrosion pit was determined by LCSM (LEXT OLS4000).

## 3. Results and Discussion

### 3.1. Microstructure

**Figure 2:**
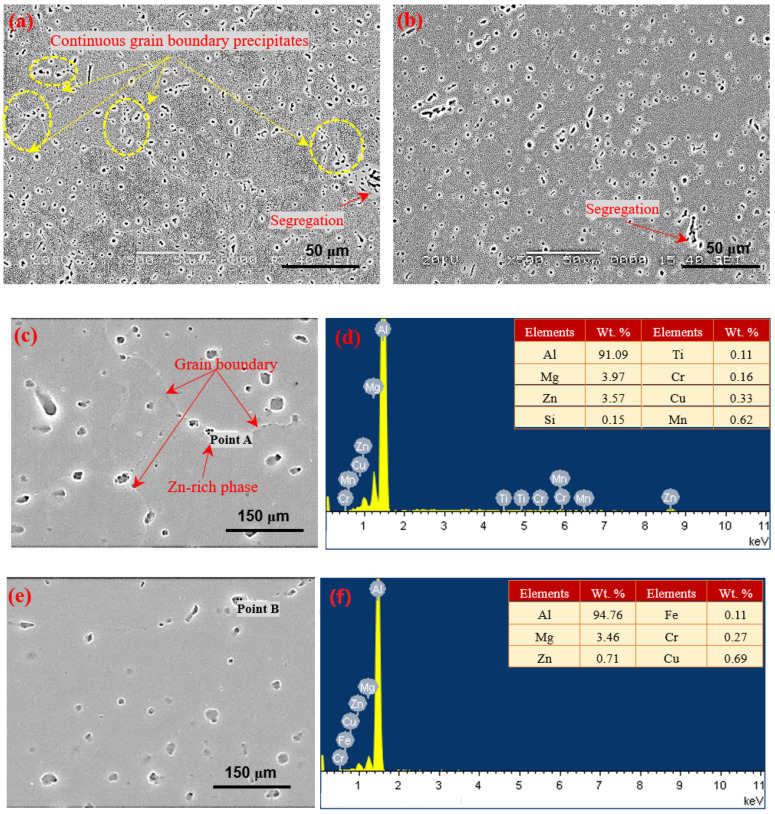
SEM and EDS results for the two kinds of weld zones, (**a**,**c**) ER5087, (**b**,**e**) ER5356, (**d**) EDS for point A, (**f**) EDS for point B.

Figure 2 shows SEM and EDS for two kinds of weld zones before electrochemical corrosion. The precipitates were distributed in a continuous network on the grain boundary, and segregation along grain boundaries also existed in the weld zone of the ER5087 welded joint shown in Figure 2a, while the precipitates and segregation were mainly dispersed in the weld zone of the ER5356 welded joint in Figure 2b. In order to further determine the composition of precipitates in the weld zones, the EDS for composition analysis was carried out. As shown in Figure 2c–f, the chemical compositions of the precipitate were Al, Mg, Zn, Fe, Cr, and Cu in the weld zone of the ER5356 welded joint. Meanwhile, there were extra trace elements of Mn and Ti in the precipitate at the grain boundary in the weld zone of ER5087 welded joint. Since the diffusion ability of Mn element was slow during the rapid cooling speed of the weld, it had no time to diffuse and thus gathered at the grain boundary and segregated along grain boundaries in the solid phase. EDS shows that the grain boundary precipitate was also Zn-rich phase in the weld zone of the ER5087 welded joint.

### 3.2. Polarization Curve

**Figure 3:**
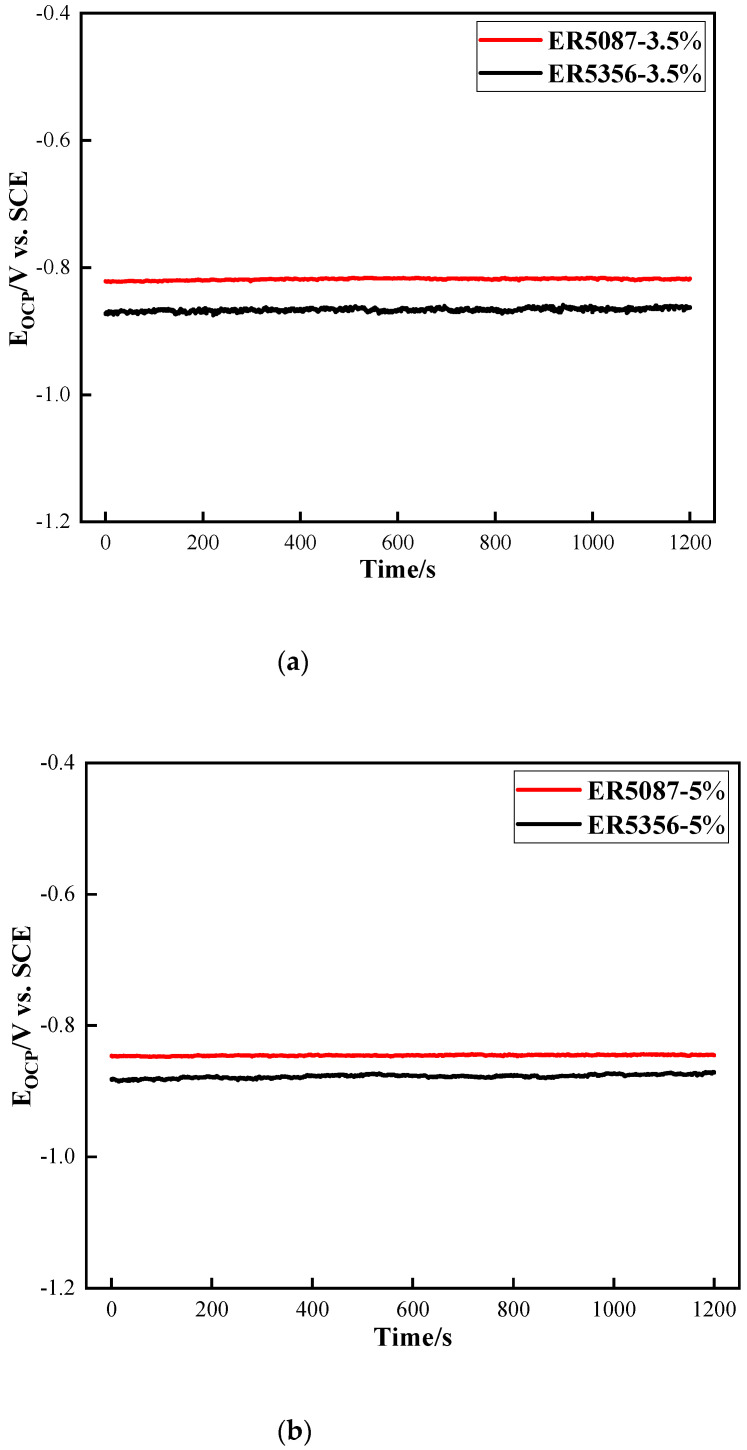
OCP of the weld zones in ER5087 and ER5356 welded joints at different concentrations of NaCl solution. (**a**) 3.5 wt.% NaCl solution, (**b**) 5 wt.% NaCl solution.

The OCP of the weld zones in ER5087 and ER5356 welded joints with 3.5 wt.% and 5 wt.% NaCl solutions, respectively, is shown in Figure 3. The OCP tended to be stable soon.

**Figure 4:**
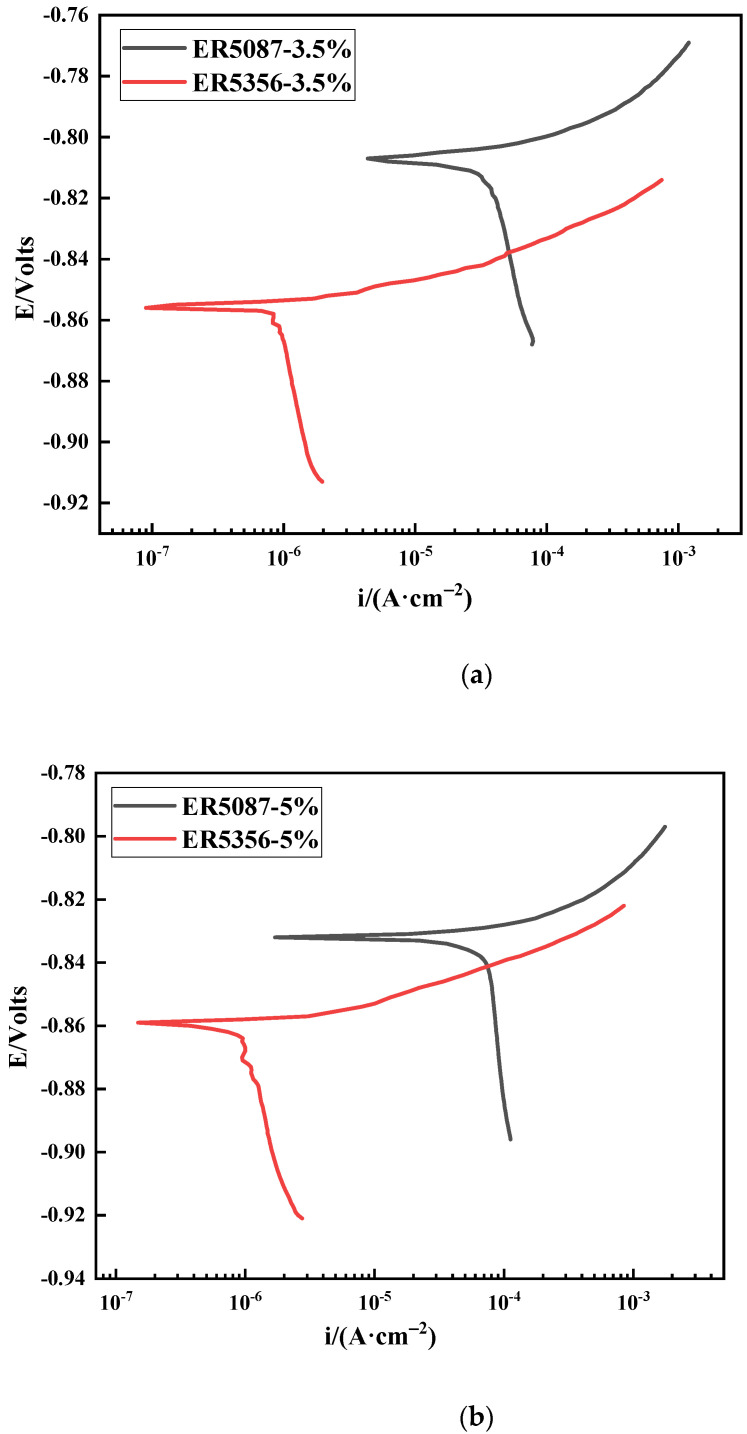
Polarization curve of the weld zones in ER5087 and ER5356 welded joints at different concentrations of NaCl solution. (**a**) 3.5 wt.% NaCl solution, (**b**) 5 wt.% NaCl solution.

Figure 4 displays the polarization curve of weld zones in ER5087 and ER5356 welded joints at different concentrations of NaCl solution. The OCP and electrochemical parameters of the weld zones in ER5356 and ER5087 welded joints are listed in Table 4. The *E*_corr_ is a thermodynamic index for estimating the tendency of electrochemical corrosion of metals under certain conditions, and a larger *E*_corr_ indicates a higher surface stability. The *I*_corr_ is a kinetic index for evaluating the corrosion degree, and a bigger *I*_corr_ represents a higher corrosion rate [15]. In the same concentration of NaCl solution, the OCP and *E*_corr_ of the weld zone in the ER5356 welded joint was slightly more negative than that in the ER5087 welded joint. The *I*_corr_ of the weld zone in the ER5087 welded joint was bigger than that in the ER5356 welded joint at the same NaCl concentration. The *I*_corr_ of the weld zone in 5 wt.% NaCl solution was bigger than that in 3.5 wt.% NaCl solution. The corrosion current density is higher. The corrosion resistance is worse. This indicated that the corrosion resistance in the weld zone of the ER5356 welded joint was better than that in the ER5087 welded joint, and the corrosion resistance of the weld zones in 3.5 wt.% solution was better than that in 5 wt.% solution.

Figure 4 shows that the cathodic polarization current was relatively low, and the anodic polarization part was not passivated. In neutral solution, the cathode part reacted as follows [16]:

(1) $$H_{2}O+2e^{-}\to H_{2}+2{\mathrm{OH}}^{-}$$

(2) $$2H_{2}O+O_{2}+4e^{-}\to 4{\mathrm{OH}}^{-}\;$$

The dissolution of the aluminum alloy electrode produced a large number of soluble metal cations in the pores. In order to achieve electrical neutrality, Cl^−^ would diffuse and enrich into the closed cell. The metal chloride in the corrosion pit would be concentrated. However, the corrosion product Al^3+^ would hydrolyze to form more stable Al(OH)_3_. The anode part underwent metal oxidation reaction:

(3) $$\mathrm{Al}\to {\mathrm{Al}}^{+3}+3e^{-}$$

(4) $${\mathrm{Al}}^{+3}+3{\mathrm{OH}}^{-}\to \mathrm{Al}{\left(\mathrm{OH}\right)}_{3}$$

The sample surface was covered by hydrogen bubbles, which resulted in relatively low cathode current density. The $\mathrm{Al}{\left(\mathrm{OH}\right)}_{3}\;$ formation of a corrosion deposition layer inhibited the external and local corrosion internal material transmission, and then formed blocked galvanic cell corrosion. Therefore, there was no passivation in the anodic polarization curve [17].

### 3.3. Electrochemical Impedance

**Figure 5:**
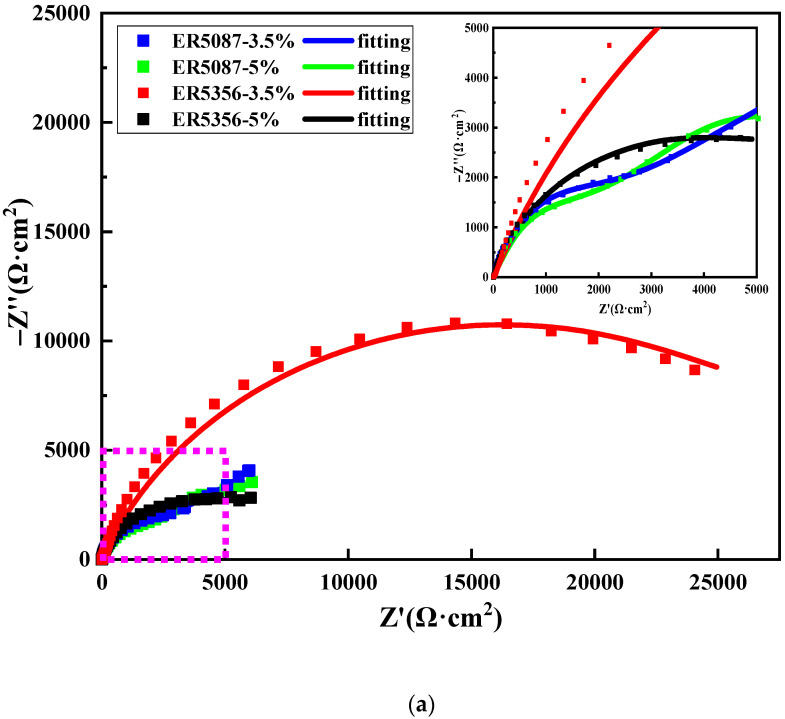

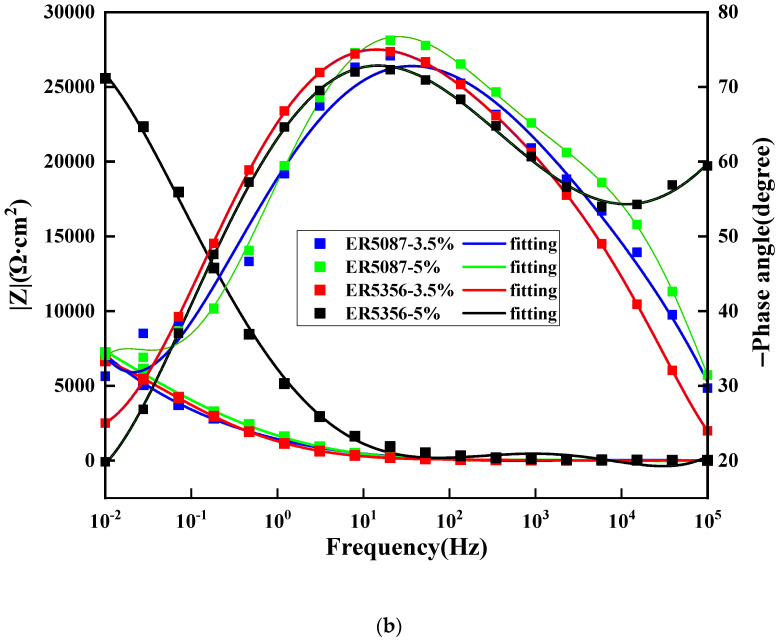
Impedance spectrum in the weld zones in ER5356 and ER5087 welded joints at different NaCl concentration (**a**) Nyquist plots, (**b**) Bode plots.

Figure 5 reveals the Nyquist plot and Bode plot of the weld zones in ER5087 and ER5356 welded joints at different concentrations of NaCl solution (3.5 wt.% and 5 wt.%). The Nyquist plot shows a compressed semicircular capacitive reactance arc of the weld zone of the ER5356 welded joint, but there was a capacitive reactance arc in the high frequency region and a straight line at a certain angle in the low frequency region for the weld zone in the ER5087 welded joint (see Figure 5a). The capacitive arc represents the new interface produced by corrosion in the corrosion process. The larger the radius of capacitive arc, the stronger the corrosion resistance of the alloy. The straight line in the low frequency region indicated the existence of Warburg impedance diffusion behavior [18,19]. The transformation process of the impedance spectrum was mainly related to the dynamic transformation in the corrosion process, and the dynamic transformation changed from transfer control to diffusion control.

Figure 5b shows the Bode diagram of the weld zones in ER5087 and ER5356 welded joints in 3.5 wt.% and 5 wt.% NaCl solutions, respectively. The phase angles were less than 90°, indicating that there was a certain difference between the ideal capacitance and the electrochemical interface of the sample. With the increase of NaCl concentration, the corresponding impedance decreased, while the phase angle increased. This was because the increase of NaCl concentration and corrosion time made the charge transfer between the alloy and the solution easier, the corrosion rate faster, and corrosion products more abundant. Finally, the intermetallic compound particles in the corrosion pit slowly separated from the aluminum matrix [20].

The radius of capacitive arc and the impedance of the weld zone in the ER5356 welded joint were bigger than that in the ER5087 welded joint. This also proved that the corrosion resistance of the weld zone in the ER5356 weld joint was better than the ER5087 welded joint.

**Figure 6:**
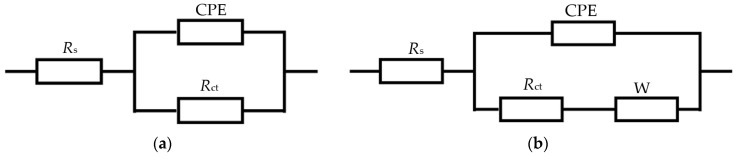
Equivalent circuit diagram of EIS fitting. (**a**) the weld zone of ER5356 welded joint, (**b**) the weld zone of ER5087 welded joint.

Figure 6 displays equivalent circuit diagram of EIS fitting. Table 5 shows The EIS electrochemical parameters of the weld zones in ER5356 and ER5087 welded joints. *R*_s_ denotes the solution resistance, *R_ct_* represents the charge transfer resistance, W and CPE are Warburg impedance and a constant phase element, respectively. CPE is related to the distribution of surface reactivity, non-uniformity, roughness, electrode porosity, and species adsorption [21]. Since there is almost no pure capacitance in the process of electrochemical corrosion, CPE is usually applied to make the fitting the electrochemical data more accurate. The impedance of CPE can be expressed by the following formula [22]:

(5) $$Z_{CPE}=\frac{1}{Y{\left(j\omega \right)}^{n}}$$

where *Y* represents the dimension of *CPE*, *j* represents the imaginary number, *ω* = 2*πf* is the angular frequency (rad/s) (*f* represents the frequency, the unit is Hz), and *n* is the index related to the diffusion effect of capacitive reactance. If *n* = 0, *CPE* represents a pure resistor, *n* = 1, a pure capacitor, and if *n* = 0.5, a Warburg impedance with diffusion characteristics.

The *R_ct_* is usually used to evaluate the sensitivity, and the smaller *R_ct_* value represents a higher corrosion current density and thus a worse corrosion resistance [23]. The *R_ct_* of the weld zones in ER5087 and ER5356 weld joints gradually decreased with the increase of NaCl concentration, indicating that the surface activity of the two kinds of weld zones increased correspondingly, and the corrosion inhibition ability decreased. This was because with the increase of NaCl concentration, the electrolyte in the solution increases, which accelerates the speed of ion exchange in the solution and reduces the *R_s_*. *n* decreased with the increase of NaCl concentration, which indicated that the smoothness of the electrode surface decreases. The increase of NaCl concentration intensified the damage of Cl^−^ to the metal matrix. Pitting corrosion occurred on the surface of the aluminum alloy and intensified the surface heterogeneity, and the dispersion effect enhanced with the increase of NaCl concentration.

Under the same NaCl concentration, the *R_ct_* of the weld zone in the ER5356 welded joint was bigger than that in the ER5087 welded joint, indicating that the corrosion resistance of the weld zone in ER5356 weld joint was better than that in the ER5087 welded joint.

The corrosion performance of weld zones mainly depends on the microstructure and electrochemical properties near the grain boundary [24,25]. The microstructure near the grain boundary mainly refers to the distribution of precipitates at the grain boundary. When the precipitates at the grain boundary were continuously distributed, a continuous corrosion channel will be formed, which will reduce the corrosion resistance of the alloy [26,27,28]. A large number of grain boundary precipitates and segregation along grain boundaries existed in the weld zone of the ER5087 welded joint. While the precipitates and segregation were mainly dispersed in the weld zone of the ER5356 welded joint (see Figure 2), so the weld zone in the ER5356 welded joint had a better corrosion resistance than that of the ER5087 welded joint.

On the other hand, the electrochemical properties of precipitates at grain boundaries and nearby micro regions will also lead to corrosion differences [29,30]. ER5356 and ER5087 welding wires are both Al-Mg alloys, Al-Mg welding wires increases the content of Mg in the weld zone and forms strengthening phase Al_8_Mg_5_ (β phase) [4,5,31]. When the content of Mg element in Al-Mg alloy exceeded 3%, it was very sensitive to stress corrosion cracking due to the precipitation of Al_8_Mg_5_ at the grain boundary, which was an anode relative to the matrix α-Al and would be preferentially corroded [32,33]. Therefore, the corrosion resistance of the weld mainly depends on the microstructure quantity as well as the size and distribution of the β-Al_8_Mg_5_ [34]. ER5087 welding wire contains more Mn element, which reduces the solubility of Mg in the matrix α-Al to make more β-Al_8_Mg_5_ precipitate phases, resulting in lower corrosion resistance.

Therefore, the electrochemical corrosion resistance of the weld zone in the ER5087 welded joint was worse than that of the ER5356 welded joint.

### 3.4. Surface Morphology after Corrosion

**Figure 7:**
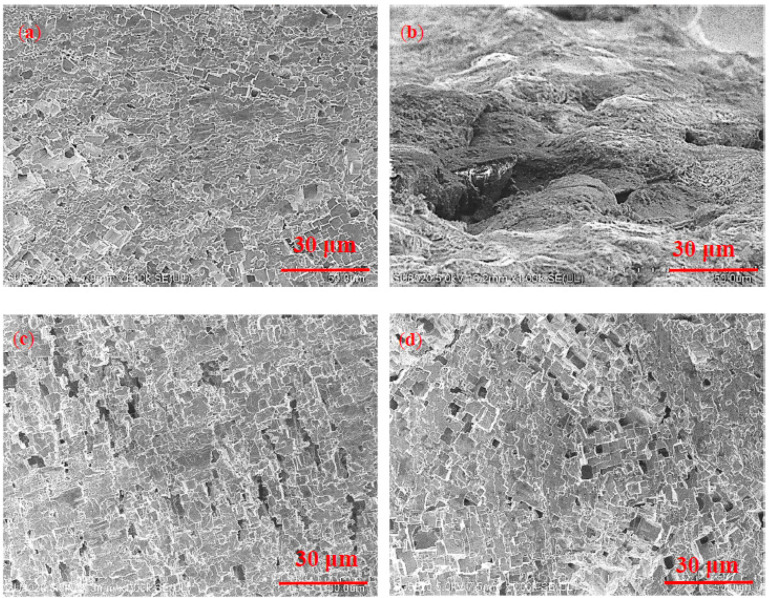
SEM for the kinds of two kinds of weld zones after electrochemical corrosion, (**a**) ER5087-3.5%, (**b**) ER5087-5%, (**c**) ER5356-3.5%, and (**d**) ER5356-5%.

Figure 7 shows the SEM of the weld zones in ER5356 and ER5087 welded joints in 3.5 wt.% NaCl and 5 wt.% NaCl solutions after electrochemical corrosion. There were many different sized pits on the surface of the samples (Figure 7a). In 5 wt.% NaCl solution (Figure 7b), the weld zone of ER5087 welded joint showed small pitting pits connected to form larger diameter pitting pits, and cracks appeared in the pitting pits. This indicated that intergranular corrosion occurs. The corrosion mechanism of the weld zone in ER5087 welded joint at 5 wt.% NaCl solution developed from pitting corrosion to intergranular corrosion. The surface pitting of the weld zone in the ER5356 welded joint in 5 wt.% NaCl solution (Figure 7d) was more severe than that in 3.5 wt.% NaCl solution (Figure 7c). There was pitting on the surface, and the pitting was not interconnected.

Compared with the weld zone of the ER5356 welded joint, the weld zone of the ER5087 welded joint contained more Zn. The higher content of Zn promoted the formation of the Zn-rich phase (see Figure 3), which was dissolved and continuously distributed along the grain boundary due to electrochemical reaction, resulting in the grain boundary becoming an anode active channel, which made the corrosion develop into the interior of the metal [35], leading to the occurrence of intergranular corrosion.

With the competitive adsorption of Cl^−^ and oxygen in NaCl solution, Cl^−^ will gradually replace OH^−^ to produce AlCl_3_ [36], leading to the formation of corrosion pits. The reaction formula is as follows [37]:

(6) $$\mathrm{Al}{\left(\mathrm{OH}\right)}_{3}+{\mathrm{Cl}}^{-}\to \mathrm{Al}{\left(\mathrm{OH}\right)}_{2}{\mathrm{Cl}}^{-}+{\mathrm{OH}}^{-}$$

(7) $$\mathrm{Al}{\left(\mathrm{OH}\right)}_{2}{\mathrm{Cl}}^{-}+{\mathrm{Cl}}^{-}\to \mathrm{Al}\left(\mathrm{OH}\right){\mathrm{Cl}}_{2}+{\mathrm{OH}}^{-}$$

(8) $$\mathrm{Al}\left(\mathrm{OH}\right){\mathrm{Cl}}_{2}+{\mathrm{Cl}}^{-}\to {\mathrm{AlCl}}_{3}+{\mathrm{OH}}^{-}$$

For the weld zone in the ER5087 welded joint at 5 wt.% NaCl solution, more Cl^−^ was adsorbed onto the active surface of weld zones, which accelerated the corrosion, resulting in the corrosion mechanism from pitting to intergranular corrosion. The corrosion products generated at the grain boundaries would expand the volume, resulting in slightly discontinuous protrusions on the metal surface, which might cause metal spalling in severe cases.

**Figure 8:**
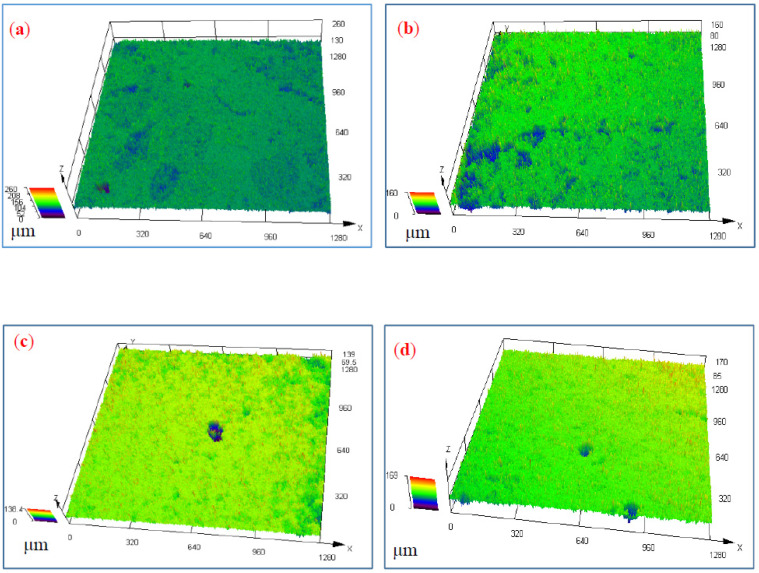
LSCM corrosion morphology for two kinds of weld zones, (**a**) ER5087-3.5%, (**b**) ER5087-5%, (**c**) ER5356-3.5%, and (**d**) ER5356-5%.

Figure 8 shows LSCM of two kinds of weld zones under different concentrations of NaCl solutions. The results show that there were some corrosion pits. The corrosion degree enhanced, and the corrosion point diffused in 5 wt.% NaCl solution. There were more corrosion pits in the weld zone of the ER5087 welded joint than ER5356 welded joint.

In order to analyze the difference of corrosion pits of the weld zones in ER5356 and ER5087 welded joints under different NaCl concentration, the max depth (*d*_max_) of corrosion pits was measured by LSCM. Table 6 shows the *d*_max_ of corrosion pit in weld zones. In the same NaCl concentration, the *d*_max_ of the corrosion pit of the weld zone in the ER5087 welded joint was bigger than that in the ER5356 welded joint. The *d*_max_ of the corrosion of the weld zone in the ER5356 welded joint with 5 wt.% NaCl was 78.5 ± 0.96 μm, which was much bigger than that with 3.5 wt.% NaCl concentration. The *d*_max_ of the corrosion pit with 5 wt.% NaCl solution was bigger than that with 3.5 wt.% NaCl solution.

## 4. Conclusions

In this study, 7N01-T4 aluminum alloy was welded by MIG with ER5087 and ER5356 welding wires, respectively. The electrochemical corrosion behavior of the two kinds of weld zones when immersed into 3.5 wt.% and 5 wt.% NaCl solution was investigated. The following conclusions can be drawn:

(1) The results of the polarization curve and EIS indicated the better corrosion resistance of the weld zone in the ER5356 welded joint compared to that in the ER5087 welded joint, which was related to the different contents of Mn and Zn elements and distribution of precipitates for the weld zones in the two kinds of welded joints.
(2) The *d_max_* for corrosion pits of the weld zone in the ER5356 welded joint was smaller than that in the ER5087 welded joint when immersed in the same NaCl concentrations. The *d*_max_ of the corrosion pit of the weld zone in the ER5356 welded joint with 5 wt.% NaCl solution was 78.5 ± 0.96 μm, which was much bigger than that with 3.5 wt.% NaCl solution. For the weld zone in the ER5087 welded joint with 5 wt.% NaCl solution, more Cl^−^ was adsorbed onto the active surface of weld zones, which accelerated the corrosion, resulting in the corrosion mechanism from pitting to intergranular corrosion.

## Figures and Tables

**Table 1:** Chemical compositions of 7N01-T4 aluminum alloy and welding wires (wt.%).

Alloy	Si	Fe	Cu	Mn	Mg	Zn	Ti	Cr	Zr	Al
7N01-T4	≤0.30	≤0.30	≤0.20	0.2–0.7	1.0–2.0	4.0–5.0	≤0.20	≤0.30	—	Bal.
ER5356	0.05	0.10	<0.01	0.14	5.00	<0.01	0.07	0.06	—	Bal.
ER5087	0.25	0.40	0.05	0.90	4.80	0.25	0.15	0.15	0.15	Bal.

**Table 2:** MIG welding process parameters of 7N01-T4 aluminum alloy.

Weld Pass No.	Welding Current/A	Welding Voltage/V	Welding Speed/(mm∙s^−1^ )
1	250	24	8
2	260	24.5	7
3	250	24	7

**Table 3:** The abbreviation of samples in this paper.

Acronyms	Notes
ER5087-3.5%	The weld zone of ER5087 welded joint in 3.5 wt.% NaCl solution
ER5087-5%	The weld zone of ER5087 welded joint in 5 wt.% NaCl solution
ER5356-3.5%	The weld zone of ER5356 welded joint in 3.5 wt.% NaCl solution
ER5356-5%	The weld zone of ER5356 welded joint in 5 wt.% NaCl solution

**Table 4:** The OCP and polarization curve electrochemical parameters of the weld zone in ER5356 and ER5087 welded joints.

Sample	OCP (mV)	*E*_corr_ (mV)	*I*_corr_ (10^−7^A·cm^−2^)
ER5087-3.5%	−817.6 ± 2	−807 ± 4	49.4 ± 0.3
ER5087-5%	−845.6 ± 1	−832 ± 3	76.5 ± 0.4
ER5356-3.5%	−863.1 ± 1.5	−856 ± 3	5.1 ± 1.2
ER5356-5%	−871 ± 2	−859 ± 2	9.8 ± 0.5

**Table 5:** The EIS electrochemical parameters of the weld zones in ER5356 and ER5087 welded joints.

Sample	*R_s_* (Ω·cm^2^)	CPE		*R*_ct_ (KΩ·cm^2^)	*Y_W_* (10^−4^ Ω^−1^·cm^−2^·s^−0.5^)
		*Y*_0_ (10^−6^Ω^−1^·cm^−2^·s^−n^)	*n* (0 < *n* < 1)		
ER5087-3.5%	2.53	57.49 ± 1.56	0.81	8.03 ± 0.23	3.44 ± 0.80
ER5087-5%	2.17	71.60 ± 1.82	0.79	7.76 ± 0.18	3.82 ± 0.78
ER5356-3.5%	5.24	20.63 ± 0.44	0.82	34.01 ± 0.16	—
ER5356-5%	3.09	74.52 ± 1.22	0.74	7.89 ± 0.21	—

**Table 6:** The *d*_max_ of corrosion pit in the weld zones.

Sample	*d*_max_ (μm)
ER5087-3.5%	71 ± 0.89
ER5087-5%	86 ± 1.20
ER5356-3.5%	53 ± 0.45
ER5356-5%	78.5 ± 0.96
